# Small nucleic acid drugs-the dawn of functional cure of chronic hepatitis B

**DOI:** 10.3389/fphar.2025.1633001

**Published:** 2025-09-26

**Authors:** Lu Zhang, Yu Cao, Sijing Zhuang, Jingjing Sun, Qiao Tong, Jianjun Xi, Shourong Liu, Rangxiao Zhuang

**Affiliations:** Hangzhou Xixi Hospital, Hangzhou, China

**Keywords:** chronic hepatitis B, RNA interference, small interfering RNA, microRNA, antisense oligonucleotides, functional cure, small nucleic acid drugs

## Abstract

Chronic hepatitis B (CHB), a chronic liver infectious disease, results from persistent hepatitis B virus (HBV) infection lasting over 6 months. It has become a substantial global public health burden. CHB is often manifested by concomitant hepatic biochemical abnormalities, and/or notable inflammatory necrosis, and/or liver histological fibrosis. If left uncontrolled, CHB can progress to severe liver diseases and may even lead to death. Although currently approved therapeutic agents can effectively suppress viral replication and, to a certain extent, reduce related complications, their ineffectiveness in targeting covalently closed circular DNA (cccDNA) fundamentally restricts their potential to achieve a clinical cure. In recent years, research focused on attaining a functional cure for CHB has been on the rise. Drugs with different targeting mechanisms and diverse therapeutic strategies have rendered a clinical cure for CHB a possibility. Among these, emerging small nucleic acid drugs show great promise, exhibiting high potential for achieving a sustained functional cure. In this review, we systematically investigate the unique structure of the HBV genome. Moreover, we delve into the classification, mechanisms of action, and pathways for small nucleic acid drugs used in CHB treatment to achieve a functional cure. Additionally, we analyze some challenges encountered in the development of these drugs and propose corresponding solutions. Furthermore, we discuss current clinical studies and combination therapies involving small nucleic acid drugs for CHB treatment.

## 1 Introduction

Chronic hepatitis B (CHB), caused by persistent hepatitis B virus (HBV) infection, remains a significant global public health challenge (World Health Organization, 2016). In April 2024, the World Health Organization released the “Global Hepatitis Report 2024” (World Health Organization, 2024). Global epidemiological data from 2022 indicate that approximately 254 million individuals were living with HBV infection worldwide. Over time, chronic HBV infection contributes to severe hepatic complications, including liver fibrosis (Zhang Q. et al., 2022; He et al., 2023), cirrhosis, and hepatocellular carcinoma (HCC) (Ringelhan et al., 2024). HBV is a non-cytopathic hepatotropic DNA virus (Tong et al., 2017; Wang et al., 2022). HBV infection can lead to diverse clinical outcomes, ranging from acute self-limiting infection to chronic persistence. The host’s age at the time of HBV exposure plays a critical role in determining disease progression (Wu and Chang, 2015). Immunocompetent adults typically develop acute, self-limited infections and eventually clear the virus (Trépo et al., 2014). In contrast, 90% of people exposed to HBV in the perinatal period develop chronic infection, and approximately 25%–30% of patients infected with HBV in infancy develop chronic infection (Hou et al., 2017). Beyond its clinical implications, chronic HBV infection imposes substantial economic burdens and psychological distress on affected individuals and their families. The current clinical management of CHB primarily involves two classes of antiviral agents: nucleoside/nucleotide analogues (NAs) (Fung et al., 2011; Hsu et al., 2023; Yang et al., 2023) and interferon-alpha (IFN-α) (Jiang et al., 2023; Zhao et al., 2024). While both drug classes can effectively suppress viral replication and mitigate liver disease progression, neither achieves complete eradication of HBV (Lee and Banini, 2019). NAs are associated with high relapse rates upon discontinuation, and prolonged use may increase the risk of drug resistance (Yang et al., 2023). IFN-α exhibits a limited immune response rate in patients and is contraindicated in cases of decompensated cirrhosis (Fung et al., 2022). Currently, the inability to effectively eliminate cccDNA and integrated HBV-DNA precludes complete or virological cure for CHB. Consequently, current clinical goals focus primarily on achieving a “functional cure” (Phillips et al., 2022; Zheng et al., 2022), defined as sustained hepatitis B surface antigen (HBsAg) loss with undetectable serum HBV-DNA (Wong et al., 2022), with or without hepatitis B surface antibody (HBsAb) seroconversion. However, residual cccDNA remains in hepatocytes, highlighting the need for novel therapeutic strategies. In response, emerging CHB therapeutics are being actively developed, targeting either direct antiviral mechanisms (inhibiting various stages of the HBV life cycle) or indirect approaches (modulating host immune responses) (Asselah et al., 2019; Xia and Liang, 2019; Maepa et al., 2021; Tang et al., 2022; Li J. et al., 2024). Among these, small nucleic acid drugs have garnered significant attention as promising candidates in pharmaceutical research.

Small nucleic acid drugs, generally refer to nucleotide sequences of no more than 30 base pairs (Chan et al., 2006), acting upstream of protein synthesis at the post-transcription and pre-translation stages (Kanasty et al., 2013; Soriano et al., 2017; Halloy et al., 2022). In contrast to conventional small-molecule drugs and monoclonal antibodies (Takakusa et al., 2023), small nucleic acid drugs are designed to target the viral post-transcriptional messenger RNA (mRNA) and pregenomic RNA (pgRNA) through the design of specific nucleotide sequences (Hui et al., 2022), This mechanism triggers gene silencing, which, in turn, effectively inhibits the production of HBV antigens and viral replication (Sun et al., 2024). As a result, it can prevent the continuous deterioration of liver damage. Unlike traditional therapeutics constrained by protein druggability requirements, small nucleic acid drugs target mRNA based on the principle of base complementary pairing, they circumvent the limitations imposed by protein druggability, thereby expanding the range of potential therapeutic targets. Furthermore, their high specificity and precision in target recognition contribute to accelerated drug development cycles. In addition, small nucleic acid drugs demonstrate significantly extended pharmacological activity compared to traditional small-molecule drugs. Through rational chemical modifications, these engineered oligonucleotides achieve enhanced metabolic stability, prolonging their plasma half-life and enabling sustained therapeutic effects with reduced dosing frequency. Although small nucleic acid drugs hold considerable promise for the treatment of hepatitis B, their translation into clinical applications remains challenging. Unmodified small nucleic acids suffer from poor stability and potential off-target effects. Current research efforts are therefore focused on addressing these limitations through advanced chemical modifications and novel delivery strategies.

In this review, we provide a comprehensive overview of the current state and future trends in the development of small nucleic acid drugs for the treatment of CHB. First, we delve into the distinctive structure of the HBV genome and explore the potential of designing small nucleic acid drugs based on this unique structure. We then introduce the main classifications and mechanisms of small nucleic acid drugs, including single-stranded antisense oligonucleotides (ASOs) and double-stranded RNAs (dsRNAs) such as small interfering RNAs (siRNAs) and microRNAs (miRNAs). Moreover, we elaborate on the pathways through which small nucleic acid drugs can achieve a functional cure. These pathways primarily involve the inhibition of viral replication and their influence on immune regulation. However, small nucleic acid drugs are highly susceptible to degradation *in vivo*, which poses numerous challenges during their development. One of the key issues is how to effectively enrich these drugs in target tissues and enable them to act on target genes. In addition to relying on appropriate chemical modifications, the precise delivery of small nucleic acid drugs using efficient delivery systems represents an important research avenue. Subsequently, we present the current research on small nucleic acid drugs for CHB treatment, covering both preclinical studies and clinical studies. We also discuss the combination therapy of small nucleic acid drugs. Finally, we have explored the future development trend of small nucleic acid drugs.

## 2 The HBV genome and the basis of drug design

**FIGURE 1:**
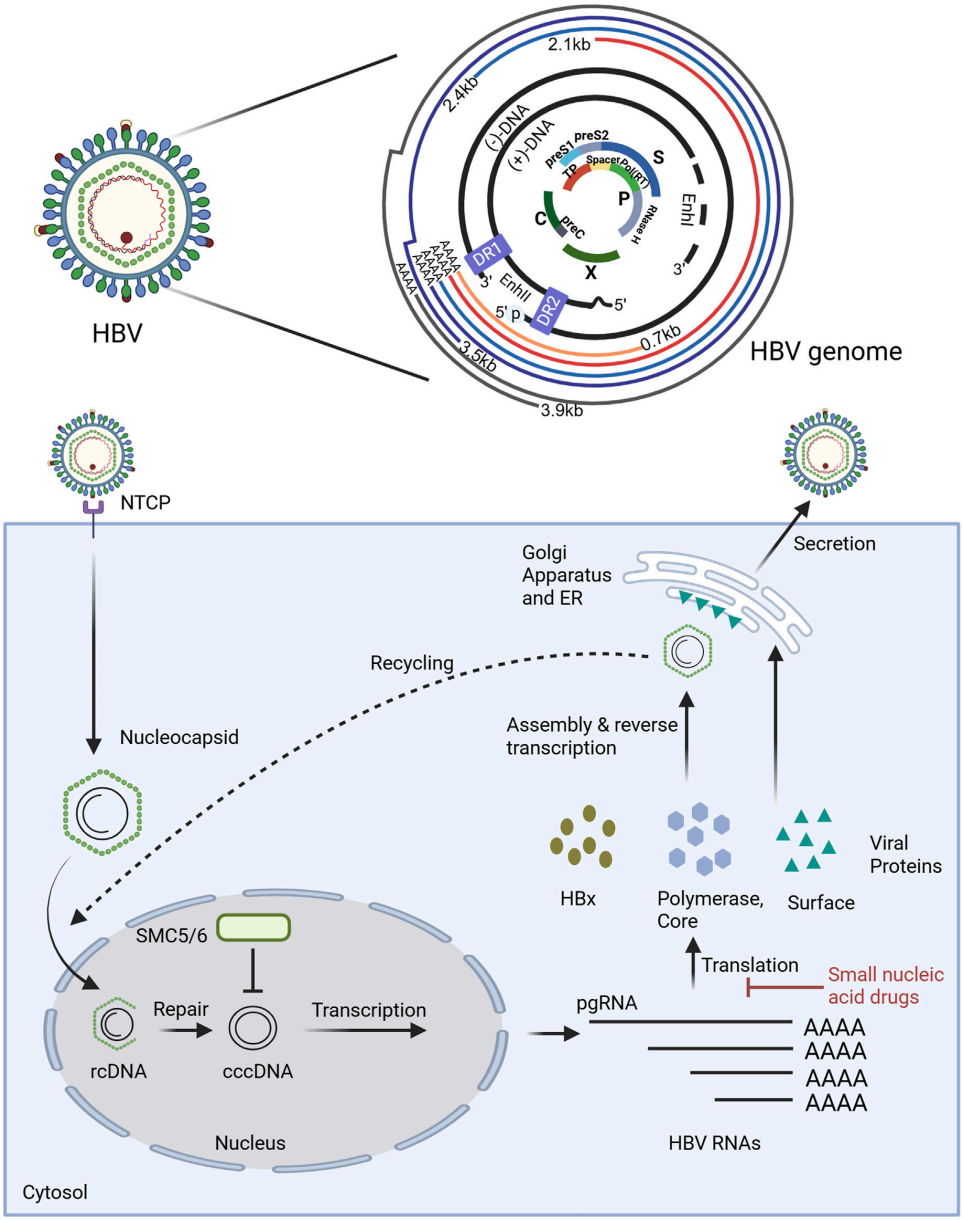
The structure of HBV genome and the mechanism of small nucleic acid drugs in the disruption of the HBV replication cycle. The HBV genome is characterized by a compact organization. As illustrated in the figure, the outer circle lines represent the viral RNA transcripts produced, all of which end in the poly-A tail. The two black lines denote the relaxed circular DNA (rcDNA) genome with a complete minus (−) strand and a incomplete plus (+) strand. The genome structure is compact. The central part shows overlapping open reading frames (ORFs), where the PreS1/PreS2/S ORF overlaps highly with the P (polymerase) ORF, and the P ORF also partially overlaps with the PreC/C ORF and X ORF. Additionally, there are some regulatory elements, the Enhancer regions I and II (EnI and EnII), and the direct repeat sequences (DR1 and DR2). The polymerase-reverse transcriptase (blue ball marked with p) covalently linked to the 5′ end of the (−) strand DNA is also shown. Small nucleic acid drugs disrupt the HBV replication cycle primarily by inhibiting the translation of viral RNAs, thereby preventing the synthesis of viral proteins essential for replication. Key host and viral factors involved in the cycle are denoted, such as NTCP (sodium taurocholate cotransporting polypeptide), SMC5/6 (structural maintenance of chromosome complex 5/6), cccDNA (covalently closed circular DNA), and pgRNA (pregenomic RNA). Illustration created by authors using BioRender.com.

HBV is a small enveloped DNA virus (Dandri, 2020), consisting of only 3200 nucleotides (nt) (Tsukuda and Watashi, 2020). The viral genome exists as a partially double-stranded relaxed circular form (rcDNA) in mature virions. Upon host cell entry, rcDNA is transported to the nucleus, where it is converted into covalently cccDNA (Nassal, 2015; Wang et al., 2016; Wei and Ploss, 2021). The episomal form persists in infected cells and serves as the transcriptional template for viral replication. Every nucleotide in HBV DNA has coding capacity, and the whole genome exhibits high informational density (Tu et al., 2017). The HBV genome comprises four overlapping open reading frames (ORFs), which are the S region, C region, P region, and X region (Michel and Tiollais, 1987) (Figure 1). Since transcription is initiated by four distinct promoters at different positions in the HBV genome, it can generate four types of polyadenylated RNAs (3.5 kb preC/C mRNA, 2.4 kb PreS1 mRNA, 2.1 kb PreS2/S mRNA, and 0.7 kb X mRNA). These transcripts are exported to the cytoplasm for protein translation and DNA replication (Tong and Revill, 2016). The 0.7 kb mRNA encodes the regulatory X protein (HBxAg), while the 2.1 kb and 2.4 kb mRNAs can encode the S, M, and L surface proteins (HBsAg), The 3.5 kb mRNA consists of two species: the longer variant, termed precore RNA, encodes the precore protein (HBeAg), whereas the shorter variant, known as pgRNA, serves as a template for reverse transcription and encodes both the core protein (HBcAg) and the P protein (HBV DNA polymerase). Despite these transcripts are initiated from different positions, they terminate at the same polyadenylation site (Weinberg and Arbuthnot, 2010). If the site is utilized as a target for small nucleic acid drug design, it is theoretically possible to simultaneously target multiple viral mRNAs, induce their degradation, and inhibit viral protein production. Moreover, if pgRNA can be targeted, it is possible to silence the HBV gene, thereby inhibiting viral protein expression and suppressing replication. However, it is worth noting that HBV gene fragments can integrate into the host genome during viral replication. This integration enables the persistence of HBsAg in the serum of patients undergoing long-term antiviral therapy, primarily derived from integrated HBV DNA fragments. Therefore, small nucleic acid drugs should also target these integrated fragments, as they retain both the complete HBsAg coding sequence and a truncated HBxAg coding region. In theory, such targeting could reduce the expression of these viral proteins (Budzinska et al., 2018; Zhang et al., 2021).

## 3 Types and mechanism of small nucleic acid drugs

**FIGURE 2:**
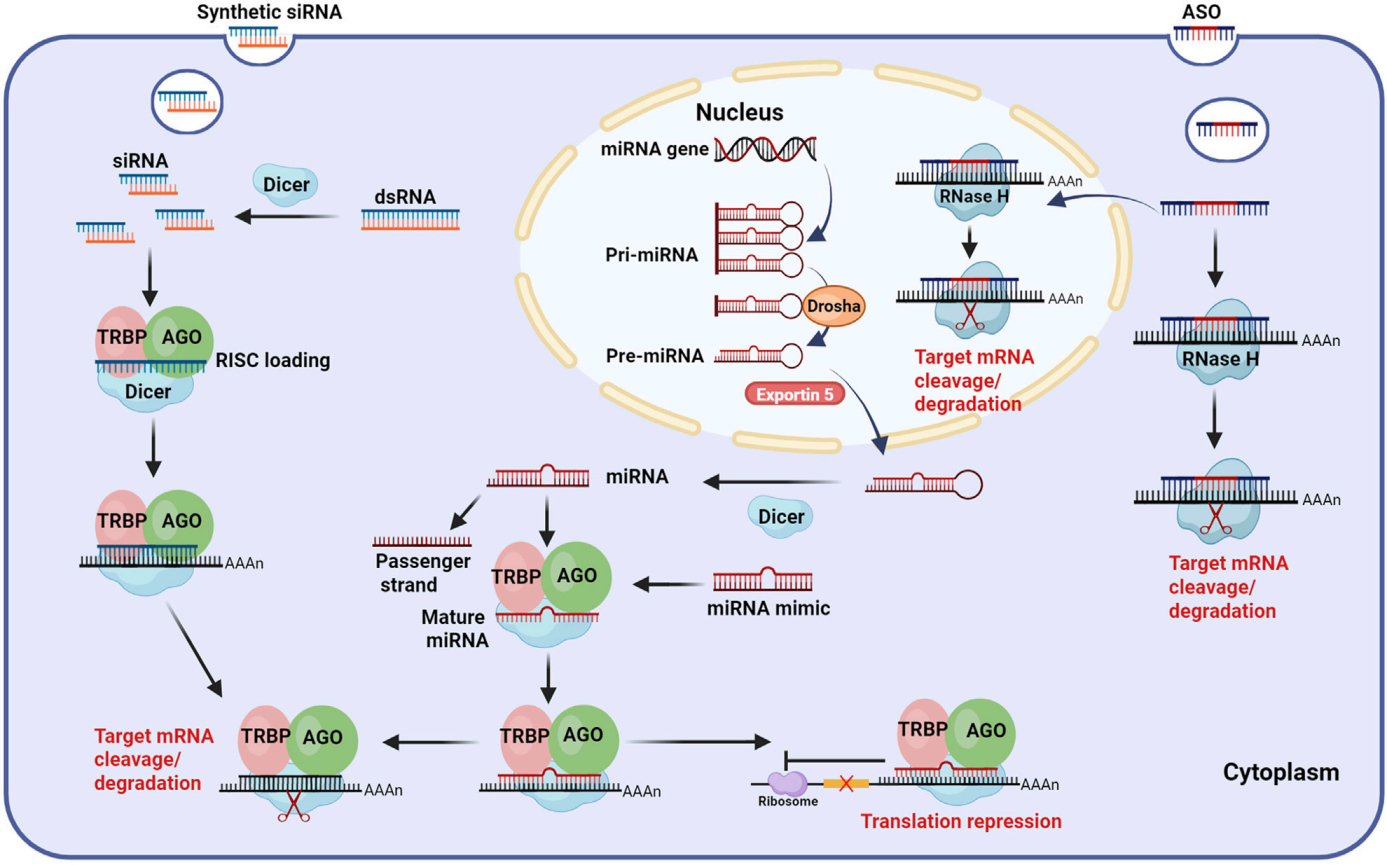
Mechanism of small nucleic acid drugs. ASO: After entering hepatocytes, ASOs (gapmer design) can bind to target mRNA with high affinity both in the nucleus and cytoplasm, forming ASO-mRNA complexes and recruiting RNase H to cleave and degrade the target mRNA. siRNA: siRNAs are derived from artificial synthesis or transcribed dsRNA, which are processed by Dicer. The processed siRNAs are loaded into RISC, where the passenger strand is discarded and the guide strand guides the active RISC to the target mRNA, binding complementarily to it, leading to the cleavage and degradation of the target mRNA. miRNA: Mature miRNA is formed by processing pri-miRNA through a series of complexes, such as Drosha, Exportin5, and Dicer. Similar to siRNA, miRNA is loaded into the RISC, where the guide strand binds partially complementary to target mRNA, ultimately leading to translation repression or cleavage and degradation of the target mRNA. Exogenous miRNA mimics can affect target mRNA through the same pathway as endogenous miRNA. Abbreviations: ASO, antisense oligonucleotide; siRNA, small interfering RNA; miRNA, microRNA; dsRNA, double-stranded RNA; Pri-miRNA, primary miRNA; Pre-miRNA, precursor miRNA; AGO, Argonaute; RISC, RNA induced silencing complex; TRBP, transactivating response RNA-binding protein; AAAA, poly-adenosine tail. Illustration created by authors using BioRender.com.

Currently, small nucleic acid drugs used for the treatment of CHB can be categorized into RNA interference (RNAi) and ASOs according to their mechanism (Figure 2).

RNAi is a unique gene regulatory mechanism mediated by dsRNAs, which induces post-transcriptional gene silencing (PTGS) and is widely observed in mammals (Tabara et al., 1998; Setten et al., 2019). This process can prevent protein synthesis by triggering the efficient and specific degradation of homologous mRNA molecules (López-Fraga et al., 2009). RNAi is highly conserved in evolution, and it plays a crucial role in gene regulation and serves as a defense mechanism against viral RNA and transposon invasion (Agrawal et al., 2003). The RNAi pathway primarily involves two classes of small RNA molecules: siRNAs and miRNAs, which exhibit biogenesis and mechanistic differences. During siRNA biogenesis, dsRNA is imported into the cytoplasm, where it associates with the RNase III enzyme Dicer and the transactivating response RNA-binding protein (TRBP). Subsequently, Dicer processes the extended dsRNA into short interfering RNA fragments (Tiemann and Rossi, 2009). The siRNA duplex consists of a guide strand (antisense strand) and a passenger strand (sense strand). The short siRNA interacts with the RNA-induced silencing complex (RISC), which is composed of several proteins, such as Argonaute 2(AGO 2, the catalytic engine of RISC) (Rand et al., 2005; Marimani et al., 2013). After binding, the passenger strand is degraded, while the guide strand directs RISC to complementary target mRNA. AGO 2 then cleaves the mRNA, leading to its degradation (Carthew and Sontheimer, 2009; Purewal and Doshi, 2024). In contrast, miRNA is generated in the nucleus, where RNA polymerase II transcribes primary miRNA (pri-miRNA). Following transcription, the nuclear microprocessor complex, consisting of the RNase III enzyme Drosha and its cofactor DGCR8 (Lee et al., 2003; Han et al., 2006; Ranganathan and Sivasankar, 2014; Medley et al., 2021), processes pri-miRNA into a hairpin-structured precursor miRNA (pre-miRNA). Pre-miRNA is then exported to the cytoplasm via Exportin5, where Dicer further trims it by removing its terminal loop into a ∼22nt miRNA duplex. Similar to siRNA, the miRNA is loaded into RISC, retaining only the guide strand. However, unlike siRNA, which requires complete Watson-Crick base pairing for target recognition, miRNA binds to mRNA through partial base pairing (Carthew and Sontheimer, 2009). It mainly achieves gene silencing by inhibiting translation rather than mRNA degradation (Bobbin and Rossi, 2016).

ASOs are single-stranded DNA, RNA, or chimeric oligonucleotides composed of a dozen to dozens of bases. Through Watson-Crick base pairing, ASOs bind to complementary mRNA sequences, thereby modulating their function (Crooke et al., 2018). ASOs exert gene regulatory effects via multiple mechanisms, primarily including target mRNA degradation, translational inhibition, and splicing modulation (Halloy et al., 2022; Hui et al., 2022; Chen et al., 2024; Miao et al., 2024). Target mRNA degradation occurs through both Ribonuclease H1 (RNase H1)-dependent and RNase H1-independent pathways. In the RNase H1-dependent mechanism, ASOs bind to target mRNAs and form the ASO-mRNA complexes (Hörberg et al., 2024), which are recognized and degraded by endogenous RNase H1. Notably, RHase H1 is distributed in both the cytoplasm and the nucleus, which allows ASOs to target not only mRNAs in the cytoplasm but also pre-mRNAs in the nucleus (Liang et al., 2017). RNase H1-independent degradation may involve alternative pathways, such as Ago2-mediated mRNA degradation, which plays an important role in RNAi regulation of mRNA degradation. Translational inhibition by ASOs primarily relies on steric hindrance. Steric-hindrance ASOs demonstrate high binding affinity to target mRNAs, and while they cannot induce mRNA degradation, they effectively interfere with RNA processing and translational functions. This interference is achieved through sequence-specific masking, which obstructs critical mRNA modification processes including splicing, polyadenylation, and ribosomal engagement. For instance, ASOs targeting the 5′cap region prevent translation initiation factors (e.g., eIF-4a) and other regulatory proteins from binding to the mRNA, thereby inhibiting translation. Similarly, ASOs complementary to upstream translation initiation codons can obstruct the binding of mRNA to protein complexes (such as ribosomal subunits), further inhibiting protein synthesis (Roberts et al., 2020). In addition, ASOs can function as splice-switching oligonucleotides (SSOs) by binding to pre-mRNA and interfering with the binding of the spliceosome to the splicing site. This modulation can lead to exon skipping or inclusion, ultimately altering gene expression (Disterer et al., 2014). Based on the aforementioned mechanism, ASOs effectively inhibit HBV replication by targeting viral RNA for degradation and suppressing gene expression. Billioud et al. (Billioud et al., 2016) demonstrated through cellular and animal experiments that ASOs directed against the highly conserved X region of HBV effectively suppress HBV RNA levels in hepatocytes. Additionally, these ASOs reduced HBsAg, HBeAg, and HBV DNA levels in cell culture supernatants and animal sera.

Although siRNAs, miRNAs, and ASOs all utilize base complementation for PTGS, they exhibit several key differences. First, their origins are different. siRNAs are typically derived from exogenous dsRNAs, whereas miRNAs are encoded by endogenous genes (Mainini and Eccles, 2020). In contrast, ASOs are synthesized artificially *in vitro*. Second, their structural features vary. siRNAs are short dsRNAs with 2 nt overhangs at the 3′end, generally 21–25 nt in length (Herbert, 2003). miRNAs, processed from hairpin-shaped pre-miRNAs, are single-stranded RNAs (ssRNAs) of approximately 19–25 nt (Kim, 2005; Liu et al., 2022). ASOs, typically 15–25 nt in length, are single-stranded oligonucleotides, often designed as gapmers (Seth et al., 2020), a structure comprising a DNA fragment in the middle and RNA wings on both sides (Aguti et al., 2024). The gapmer design not only enhances ASO binding affinity to target mRNA but also reduces its degradation by nucleases. Third, their sites of action differ. siRNAs function in the cytoplasm, whereas miRNAs undergo nuclear processing before entering the cytoplasm. ASOs can play a role in both the nucleus and the cytoplasm. Fourth, their cellular uptake mechanisms are different. Both siRNAs and miRNAs require carrier-mediated delivery (Torres-Vanegas et al., 2021; Vaillant, 2022), whereas ASOs can enter hepatocytes in carrier-bound or free form. Binding with the carrier can increase the targeting ability to hepatocytes, reduce systemic exposure, and lower toxic side effects. Finally, ASOs generally exhibit a shorter half-life than siRNAs and miRNAs due to their mechanism of directly binding to and blocking the translation of target mRNAs. In contrast, siRNAs and miRNAs can be recycled *in vivo*, and the half-life can be up to several months (Revia et al., 2019).

## 4 Pathways for achieving functional cure

Current challenges in achieving a functional cure for hepatitis B include the persistence of cccDNA reservoir within hepatocyte nuclei that evade complete eradication, as well as the integrated HBV DNA fragments in the host genome that sustain residual viral replication. Furthermore, chronic HBV infection induces immune tolerance and functional impairment of the host immune system, resulting in impaired host antiviral immune responses (Gane, 2020). Therefore, a functional cure necessitates dual strategies: suppression of viral replication and restoration of the host immune system. Small nucleic acid drugs exhibit unique advantages in addressing both aspects.

In terms of antiviral effects, small nucleic acid drugs exhibit a direct anti-HBV effect. These drugs are capable of degrading or silencing all viral mRNAs transcribed from cccDNA and integrated HBV DNA. This mechanism enables them to exert a potent and highly specific inhibitory action on the synthesis of HBV antigens. Concurrently, they effectively impede the replication of HBV DNA.

Simultaneously, small nucleic acid drugs have an important indirect influence on modulating the host’s immune response. In contrast to therapeutic vaccines or interferons, which directly stimulate the immune system, small nucleic acid drugs primarily function by effectively reducing the viral antigen load, particularly HBsAg. This reduction helps alleviate antigen-mediated immune suppression in the host. Prolonged exposure to high levels of viral antigens can have a detrimental impact on HBV-specific immune cells, such as T cells and B cells. Specifically, it leads to a decrease in the number and a decline in the function of virus-specific T cell clones. Although the decline rate of B cells is not as pronounced as that of T cells, the effect of antigen levels on B cells remains substantial (Wooddell et al., 2021). Small nucleic acid drugs can effectively inhibit HBV viral antigens. This not only offers an opportunity for the recovery or reconstruction of the host’s immune response but also enhances the host’s anti-HBV immune activity to a certain degree (Hui et al., 2024). Michler et al. demonstrated in mice with ongoing HBV replication that small nucleic acid drugs have immunomodulatory effects (Michler et al., 2020). They combined siRNA with a therapeutic vaccine. The results showed that siRNA effectively decreased HBsAg levels, increased the quantity and improved the function of HBV-specific CD8^+^ T cells in mice, and raised the levels of HBV-neutralizing antibodies. The “unlocking” effect of small nucleic acid drugs on the host immune system is of fundamental importance for achieving a functional cure for CHB.

## 5 Challenges and strategies

Compared to small-molecule and monoclonal antibody drugs, small nucleic acid drugs exhibit superior targeting ability and specificity. They can be designed for targets previously deemed “undruggable” by the other two drugs (Crooke et al., 2021). However, the research process for small nucleic acid drugs is fraught with numerous challenges (Whitehead et al., 2009). First and foremost, maintaining the integrity and stability of small nucleic acid drugs is a formidable task. Unmodified or naked small nucleic acid drugs, particularly ASOs, have a short half-life. In the *in vivo* environment, they are highly susceptible to enzymatic degradation, chemical degradation (such as oxidation and hydrolysis), or clearance by the kidneys (Wittrup and Lieberman, 2015). Moreover, exogenous small nucleic acid drugs are recognized by the immune system. This recognition triggers an immune response, which can destroy the nucleic acid structure (Zhang Z. et al., 2022). In addition, the off-target effects of small nucleic acid drugs pose a significant risk. They may lead to unintended gene activation or silencing, potentially resulting in severe adverse reactions. Insufficient targeting often leads to suboptimal drug concentrations at the target tissues. To achieve the desired therapeutic effect, higher doses are required. However, increased dosages raise more safety concerns (Jackson and Linsley, 2010; Sun et al., 2024). Finally, the high molecular weight and negative charge of small nucleic acid drugs present a major obstacle. The negatively charged lipid bilayer of the cell membrane acts as a barrier, making it difficult for these drugs to penetrate. Consequently, cells struggle to take up small nucleic acid drugs effectively (Stoddard et al., 2018). Even if the drug enters the cell successfully, it faces the problem of being captured by endosomes. Once captured, it is transported to lysosomes for degradation. Only drugs that successfully escape from endosomes can become active and exert therapeutic effects (Miao et al., 2024). All these factors impede the ability of small nucleic acid drugs to reach the nucleus and act on target genes. In response to these issues, two key technologies have emerged in recent years: chemical modification and delivery systems (Friedrich and Aigner, 2022; Ali Zaidi et al., 2023), which have promoted the development and clinical application of small nucleic acid drugs.

### 5.1 Chemical modification

**FIGURE 3:**
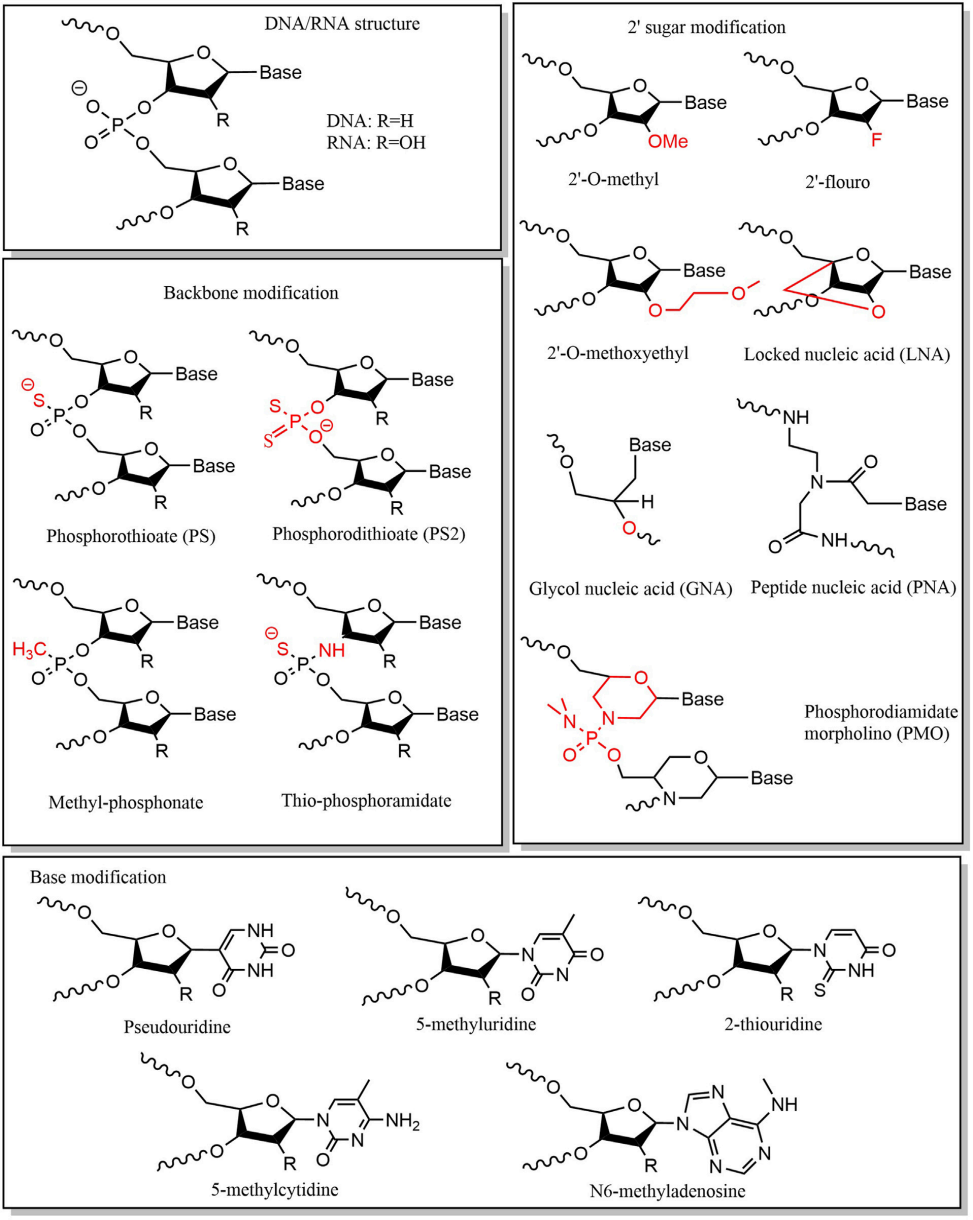
Chemical modification stretegies.

Small nucleic acid drugs consist of three fundamental structural components: phosphate groups, ribose, and base. Chemical modifications of these components can enhance stability, minimize off-target effects, and reduce immunogenicity (Yu et al., 2009; Prakash, 2011; Liu et al., 2025). Chemical modification of the phosphate group is mainly achieved by modifying the structure of phosphorus atoms on the phosphate backbone, which is a key factor affecting the stability of nucleic acids. A widely adopted strategy is substituting the nonbridging oxygen with sulfur, forming phosphorothioate (PS) linkages. This modification enhances nuclease resistance, making it a common feature in ASOs. (Eckstein, 2014; Shen et al., 2019). However, PS modifications may reduce binding affinity to target sequences (Khvorova and Watts, 2017), necessitating careful optimization of sulfur substitution positions and frequency. Typically, PS modifications are introduced at the end of the sequence (Friedrich and Aigner, 2022). The PS linkages exhibit two stereoisomeric configurations: Sp and Rp. Iwamoto et al. demonstrated that the stereoisomerization of the PS linkages affects the therapeutic efficacy of ASOs (Iwamoto et al., 2017), with the Sp-configured PS linkages conferring greater stability than Rp, prolonging ASO activity *in vivo*. Alternative phosphate modifications include replacing oxygen with alkyl or amine groups or substituting the entire phosphate group with amides or alkoxy groups (Martínez-Montero et al., 2023). Ribose modifications primarily target the 2′-hydroxyl (2′-OH) group (Le et al., 2021). Common modifications include: 2′-methoxy (2′-OMe), 2′-oxy-methoxyethyl (2′-MOE), and 2′-fluoro (2′-F) (Wu et al., 2014; Lim et al., 2021; Friedrich and Aigner, 2022; Holm et al., 2022; Jin and Zhong, 2023). The 2′-OH group is a key site for enzymatic hydrolysis, and its modification can protect nucleic acids from ribonuclease attack. More extensive modifications involve altering the ribose ring structure, including: locked nucleic acids (LNAs), unlocked nucleic acids (UNAs), glycol nucleic acids (GNAs), tricyclic DNA, peptide nucleic acids (PNAs) and phosphorodiamidate morpholino oligomers (PMOs) (Zhou et al., 2008; Campbell and Wengel, 2011; Kafil et al., 2022; Yao et al., 2024). These modifications enhance thermal stability, hybridization affinity, and *in vivo* performance. Small nucleic acid drugs can also affect drug efficacy through base modification. Base modification and base replacement can largely reduce immune recognition and improve stability, with common substitutions including: 5-position substitution of pyrimidine and the 8-position substitution of purine. The commonly used types of base modification are pseudouridine, 5-fluorouracil, 5-iodouracil, 2-thiouridine, N1-methyl-pseudouridine, 5-methyluridine, 5-methoxyuridine, 5-methylcytidine, N6-methyladenosine, and so on (Anderson et al., 2010; Wahba et al., 2011; Valenzuela et al., 2014; Helm et al., 2022; Qu et al., 2024) (Figure 3).

Currently, small nucleic acid drugs in development or clinical use predominantly employ strategic combinations of chemical modifications. For instance, bepirovirsen—an investigational ASO for subcutaneous administration in CHB—adopts a gapmer architecture. The first and last five nucleotides of its sequence are modified with 2′-MOE, and the middle segment is uniformly modified with PS. This dual-modification approach confers enhanced enzymatic stability (Han et al., 2022), increased plasma protein binding affinity, and reduced renal clearance. Additionally, the central unmodified DNA gap region retains high RNase H recruitment efficiency. The gapmer design for ASO thus optimizes both target binding capacity and pharmacokinetic stability (Dindot et al., 2023).

### 5.2 Delivery system

Although chemical modifications have enhanced the stability of small nucleic acid drugs, significant challenges remain unresolved. The development of delivery systems has notably improved the targeted delivery of these therapeutics. Currently, small nucleic acid drug delivery systems are broadly categorized into two major classes: non-viral vectors and viral vectors (Paunovska et al., 2022). Among these, non-viral vectors offer wider applicability and can be further subdivided into ligand-coupled conjugates and nanocarrier-based delivery systems. Conjugate-mediated delivery strategies mainly include N-acetylgalactosamine (GalNAc) conjugates (Springer and Dowdy, 2018; Thangamani et al., 2021), Lipid conjugates (Biscans et al., 2019; Biscans et al., 2022), antibody and peptide conjugates (Sela et al., 2023), etc. Alternatively, nanocarrier-based systems encapsulate small nucleic acids within nanostructured vehicles, improving bioavailability and biodistribution. Widely studied nanocarriers include: lipid nanoparticles (LNPs) (Samaridou et al., 2020; Jung et al., 2022) and polymeric nanocarriers (Miyata, 2016).

GalNAc and its derivatives have emerged as a promising platform for liver-targeted delivery of small nucleic acid drugs due to their strong liver-targeting ability (Thangamani et al., 2021). The liver targeting ability of GalNAc is based on its high affinity for the asialoglycoprotein receptor (ASGPR), a specific endocytosis receptor on the surface of hepatocyte membranes (Huang, 2017; Nguyen et al., 2024; Ruchi et al., 2024). When GalNAc-conjugated small nucleic acid drugs are endocytosed into the hepatocyte, the chemical bond connecting GalNAc and the small nucleic acid drug undergoes pH-sensitive degradation (Ranasinghe et al., 2022), allowing the small nucleic acid drug to escape from endosomes and enter the cytoplasm to exert its active effects. This mechanism underpins the clinical development of multiple GalNAc-conjugated therapies for CHB, such as AB-729 (An, 2023), RG-6346, ALG-020572 (Hui et al., 2022), VIR-2218 (Gupta et al., 2021), and JNJ-3989 (Debacker et al., 2020; Gane et al., 2020). While GalNAc conjugation enhances hepatocyte-specific delivery, it may compromise therapeutic efficacy in certain contexts. GlaxoSmithKline attempted to modify bepirovirsen with GalNAc, developing the new drug GSK3389404. However, Clinical trials revealed inferior HBsAg suppression compared to the parental compound (Agarwal et al., 2022), and eventually, the clinical study on GSK3389404 was terminated in phase II. Mechanistic studies attribute this limitation to altered biodistribution: unconjugated bepirovirsen primarily accumulates in Kupffer cells, whereas GalNAc-conjugated bepirovirsen (GSK 3389404) mainly accumulates in hepatocytes. Therefore, GalNAc conjugation may enhance hepatocyte uptake but restricts GSK3389404 uptake in hepatic sinusoidal and Kupffer cells, ultimately attenuating immune signaling and overall antiviral activity (Prakash et al., 2014).

Moreover, LNP-based delivery systems are an ideal option for delivering small nucleic acid drugs. LNPs are spherical carriers with a diameter of roughly 100 nm, composed of ionizable lipids, auxiliary lipids (such as phospholipids), cholesterol, and polyethylene glycol-modified lipids (PEGylated lipids) (Cheng and Lee, 2016; Kulkarni et al., 2019). The core constituent of LNPs is the ionizable lipid, which is characterized by its ability to alter its charged properties in accordance with the pKa of the lipids and the ambient pH values (Cullis and Felgner, 2024). Given that the majority of ionizable lipids possess a hydrophilic N-terminus, they become positively charged through binding with hydrogen ions under acidic conditions (pH < 6.0) and remain uncharged under neutral conditions. As a result, LNPs prepared with ionizable lipids can engage in electrostatic interactions with small nucleic acid drugs under acidic conditions, leading to high encapsulation efficiency. Under physiological conditions (pH 7.4), they are uncharged, which safeguards the structural integrity of the LNPs and minimizes toxic side effects (Kulkarni et al., 2018). Once the LNPs are endocytosed into the endosome, the acidic environment induces protonation of the ionizable lipids. This prompts electrostatic interactions between the LNPs and the endosomal membrane, causing membrane disruption and the subsequent release of small nucleic acid drugs. Owing to their outstanding biocompatibility and stability, LNPs are currently extensively utilized in the development of small nucleic acid drugs. Arbutus Biopharma has developed two RNAi drugs, ARB-1467 and ARB-1740, for the treatment of CHB using LNP delivery technology. ARB-1467 is an LNP formulation that contains three siRNAs. These siRNAs can simultaneously target three distinct sites in the HBV genome, thereby achieving post-transcriptional inhibition of HBV proteins. ARB-1740 utilizes the same LNP delivery technology as ARB-1467 but employs different RNAi trigger molecules (Thi et al., 2018). Regrettably, both ARB-1467 and ARB-1740 were terminated at the clinical Phase II stage due to suboptimal efficacy.

## 6 Experimental small nucleic acid drugs development for the treatment of CHB

Given that HBV mRNA plays an important role in HBV viral replication, small nucleic acid drugs capable of either degrading mRNA or impeding its translation are regarded as the most promising contenders for attaining a functional cure. At present, several small nucleic acid drugs for CHB treatment have entered Phase II/III clinical trials (Table 1). It is reasonably expected that an increasing number of small nucleic acid drugs will commence their development journey in the foreseeable future.

**TABLE 1:** Development of small nucleic acid drugs for CHB.

Type	Agent	Stage of development	Chemical modification and delivery system	Company
ASO	Bepirovirsen (GSK-3228836)	Phase III	2′-MOE (gapmer)	Ionis Pharma, USA with GSK
	AHB-137	Phase II	Naked ASO	AusperBio, China
	GSK-3389404	Phase II discontinued	GalNAc	GSK Biologicals, UK
	RO-7062931 (RG-6004)	Phase I discontinued	GalNAc and LNA	Roche, Switzerland
	ALG-020572	Phase I discontinued	GalNAc	Aligos Therapeutics, USA
	ALG-021682	Preclinica	GalNAc and GuNA	Aligos Therapeutics, USA
	ALG-021639	Preclinica	GalNAc and BNA	Aligos Therapeutics, USA
siRNA	Elebsiran (BRII-835, VIR-2218)	Phase II	ESC+ and GalNAc	Alnylam and VirBiotech, USA with Brii Biosciences, China
	GSK5637608 (JNJ-3989)	Phase II	GalNAc	GSK Biologicals, UK
	AB-729	Phase II	GalNAc	Arbutus Biopharma, USA
	RBD1016	Phase II	GalNAc	Ribo life science, China
	HRS-5635	Phase II	GalNAc	Hengrui Pharmaceuticals, China
	TQA3038	Phase II	GalNAc	ChiaTai TianQing, China
	Xalnesiran (RO-7445482, RG-6346)	Phase II discontinued	GalNAc	Roche with Dicerna, USA
	ARC-520	Phase II discontinued	Cholesterol-conjugated and DPC technology	Arrowhead Pharma, USA
	ARB-1467	Phase II discontinued	LNP	Arbutus Biopharma, USA
	ARB-1740	Phase II discontinued	LNP	Arbutus Biopharma, USA
	BW-20507	Phase I/II	GalNAc	Argo Biopharma, China
	HT-101	Phase I	GalNAc	Hepa Thera Bio., China
	ALG-125755	Phase I	GalNAc	Aligos Therapeutics, USA
	SA011	Preclinica	GalNAc	Suzhou Siran Biotechnology Co., Ltd., China
	SA012	Preclinica	GalNAc	Suzhou Siran Biotechnology Co., Ltd., China
	OLX703A	Preclinica	GalNAc	Olix Pharmaceuticals, South Korea with Pharmaron, China
	STP155G	Preclinica	PDoV-GalNAc	Sirnaomics, USA
	ALG-072571	Preclinica	GalNAc PD-L1 siRNA	Aligos Therapeutics, USA
	KW-040	Preclinica	GalNAc	Kawin Technology, China with Anlong Bio, China

### 6.1 ASO

To date, several ASO drugs have advanced into clinical trials. Among them, bepirovirsen (Lens, 2023) and AHB-137 have demonstrated remarkable potential in the realm of CHB treatment.

GSK-3228836 (bepirovirse), an ASO, is a collaborative development of Ionis Pharmaceuticals Inc. and GlaxoSmithKline Pharmaceuticals. It has successfully concluded its clinical Phase II trial and embarked on a Phase III clinical study in 2023. The full-length sequence of bepirovirsen is GCA​GAG​GTG​AAG​CGA​AGT​GC, which is designed to target the overlapping region of the X protein and P protein ORFs (Yuen et al., 2021). This strategic targeting enables it to act on all HBV mRNAs, encompassing transcripts from integrated HBV DNA and pgRNAs, thereby effectively inhibiting HBV replication. Moreover, bepirovirsen promotes the clearance of HBV by triggering the innate immune response. It achieves this by activating Toll-like receptor 9 (TLR9) (Vaillant, 2023). After the successful completion of Phase I and IIa clinical trials, bepirovirsen commenced a Phase IIb clinical trial (NCT04449029) in 2020. The Phase IIb trial enrolled 457 participants from 123 centers across 22 countries or regions. Among them, 230 participants had never received NA treatment, while 227 were on stable NA treatment. The participants were randomly divided into four groups at a ratio of 3:3:3:1 to evaluate the efficacy and safety of bepirovirsen after 12-week and 24-week treatment courses. The primary treatment endpoint of this Phase IIb trial was to achieve a reduction in HBsAg and HBV DNA levels below the limit of detection after 24 weeks of treatment, in the absence of any other new treatment. Yuen et al. reported the data from the Phase IIb clinical trial (Yuen et al., 2022b). The results indicated that among patients treated with 300 mg of bepirovirsen for 24 weeks, 9% of those receiving combination NA therapy and 10% of those receiving a single agent therapy reached the primary treatment outcome. Additionally, the findings suggested that baseline HBsAg levels might predict treatment efficacy. Patients with lower baseline HBsAg levels were more likely to achieve the primary outcome event. After 24 weeks of treatment with 300 mg bepirovirsen, among patients with baseline HBsAg levels <3,000 IU/mL, 12% of those receiving NA therapy and 25% of those not receiving NA therapy achieved the primary endpoint, which was consistent with the results of the Phase IIa study. During the initial 12 weeks of the trial, bepirovirsen was associated with a higher incidence of adverse events compared to the placebo. The most frequently reported adverse event was injection site reactions, and no treatment-related deaths were reported, demonstrating that bepirovirsen has an acceptable safety profile. Currently, the Phase III clinical trial of bepirovirsen is in progress. The objective of this trial is to further confirm its efficacy on a larger scale and offer a novel treatment option for patients with CHB.

AHB-137, a non-coupled ASO jointly developed by Ausper Biopharma Co., Ltd. and AusperBio Therapeutics, Inc., is presently undergoing Phase II clinical trials. At the 2024 annual meeting of the European Association for the Study of the Liver (EASL, 2024), researchers disclosed partial data from two studies. One was an international multicenter Phase I clinical trial of AHB-137 (AB-10-8001, NCT05717686), and the other was a Phase I/IIa clinical study carried out in mainland China (AB-10-8002, NCT06115993) (Gane, 2024; Wang, 2024). The outcomes indicated that following a 4-week treatment course of AHB-137, 12% (5 out of 40) of CHB patients achieved sustained undetectable HBsAg levels (below the lower limit of quantification, 0.05 IU/mL). Moreover, two patients experienced seroconversion, as evidenced by the detection of HBsAb. Subsequently, at the 2024 annual meeting of the American Association for the Study of Liver Diseases (AASLD2024) (Ding et al., 2024), researchers presented the preliminary results of the clinical trial (NCT06115993). The results revealed that by week 12, 62% (20 out of 32) of participants in the 300 mg treatment group and 43% (10 out of 23) in the 225 mg treatment group had achieved HBsAg seroclearance. Notably, the majority of these seroclearance events occurred within the first 8 weeks of treatment, accounting for 44% and 30% in the 300 mg and 225 mg treatment groups, respectively. In the 300 mg treatment group, 50% (7 out of 14) of participants with baseline HBsAg levels ranging from 1,000 IU/mL to 3,000 IU/mL achieved HBsAg seroclearance. Among the 30 participants who achieved HBsAg seroclearance, 47% had experienced seroconversion by week 12, as manifested by the detection of HBsAb levels exceeding 10 mIU/mL. In addition, AHB-137 exhibited good safety and tolerability profiles. There were no reported serious adverse events (SAEs), and no participants discontinued the treatment. The most prevalent treatment-related adverse events (TRAEs) were of grade 1–2 severity, with injection site reactions being the most common.

### 6.2 siRNA

The advancement of RNAi technology has given impetus to the development of siRNA drugs. At present, pharmaceutical companies globally have formulated a diverse array of candidate siRNA drugs for CHB. Notably, a substantial number of these drugs have exhibited potent inhibitory impacts on the expression of viral proteins essential for HBV replication.

VIR-2218 (elebsiran, BRII-835), a siRNA drug, has been specifically developed for the treatment of CHB. This innovative drug utilizes the Enhanced Stabilization Chemistry plus (ESC+) technology (Gane et al., 2023). ESC+ technology involves strategic modifications with PS, 2′-OMe, and 2′-deoxy-2′-F. To precisely target the liver, VIR-2218 is equipped with a triantennary GalNAc delivery system. The drug is designed to target the conserved X region of the HBV genome. By doing so, it aims to silence all HBV transcripts, encompassing cccDNA and integrated HBV DNA. Remarkably, VIR-2218 exhibits pharmacological activity against all 10 HBV genotypes (from A to J) (Gupta et al., 2021; Lim et al., 2022b; Schlegel et al., 2022; Gane et al., 2023). The VIR-2218-1001 (NCT03672188) trial is a Phase I/II randomized, double-blind, placebo-controlled study focusing on VIR-2218. In the second part of this study, the impact of different doses of VIR-2218 was evaluated in non-cirrhotic participants with either HBeAg-negative or HBeAg-positive chronic HBV (cHBV) infection. A total of twenty-four patients were administered two subcutaneous injections of elebsiran at 4-week intervals, with doses of 20 mg, 50 mg, 100 mg, or 200 mg. The results showed that, compared to the placebo group, patients in the various dosage groups experienced a reduction in HBsAg levels, and this reduction was dose-dependent. Higher doses of elebsiran were more effective in suppressing HBsAg. Specifically, 50% (12 out of 24) of the patients treated with elebsiran had their HBsAg levels drop below 100 IU/mL. The 200 mg elebsiran group achieved a maximum reduction of 1.65 log IU/mL. Although no participant achieved HBsAg seroclearance or seroconversion during the 48-week follow-up period, it is hypothesized that elebsiran may play a crucial role in the functional cure of CHB when used in combination with other drugs. HBsAg level below 100 IU/mL is significantly associated with the likelihood of HBsAg clearance(Moini and Fung, 2022). In another open-label Phase II study (Lim et al., 2022a), researchers compared two dosing regimens of elebsiran: one with 2 doses and the other with 6 doses of 200 mg administered subcutaneously every 4 weeks. The results indicated that the 6-dose regimen led to a greater average maximum reduction in HBsAg and a longer duration of HBsAg reduction. Both dosing regimens demonstrated similar safety and tolerability profiles.

JNJ-3989 (JNJ-73763989, GSK5637608, daplusiran/tomligisiran) is an siRNA drug developed by Arrowhead Pharmaceuticals for the treatment of CHB. This innovative siRNA drug contains two siRNA triggers specifically designed to target the HBV S and X ORFs (Gane et al., 2022). It can achieve PTGS of all HBV RNAs, including those transcribed from cccDNA and integrated HBV DNA. Each siRNA in JNJ-3989 is conjugated with a triantennary GalNAc, which is a key feature ensuring efficient delivery of the drug to the liver. In a Phase IIa open-label trial (NCT03365947, AROHBV1001), researchers evaluated the safety and tolerability of multiple ascending doses of JNJ-3989 in patients with CHB (Yuen et al., 2022c). The study included two groups of patients: those who had previously received NA treatment and those who had not. These patients were administered JNJ-3989 at doses of 25 mg, 50 mg, 100 mg, 200 mg, 300 mg, or 400 mg, with dosing intervals of once a week (QW), every 2 weeks (Q2W), and every 4 weeks (Q4W). Throughout the entire study period, all patients continued their NA therapy. The results indicated that all Q4W dose groups showed a consistent decline in HBsAg levels from day 0 to day 112. Moreover, the degree of HBsAg reduction was found to be dose-correlated. When the dose was in the range of 25–100 mg, higher doses of JNJ-3989 were associated with more significant reductions in HBsAg. However, when the dose exceeded 100 mg, the HBsAg levels reached a plateau. Among patients receiving JNJ-3989 at doses of 100 mg or higher with a Q4W dosing schedule, 97.5% (39 out of 40) achieved ≥1 log10 IU/mL HBsAg reduction. By day 112, 75% (30 out of 40) of these patients had HBsAg levels below 100 IU/mL. Another significant finding was that more frequent dosing intervals did not have an impact on either the magnitude or the rate of HBsAg reduction.

AB-729 (imdusiran), a GalNAc-conjugated siRNA drug, is designed with a single siRNA trigger. This trigger is targeted at all HBV transcripts, effectively inhibiting the production of all HBV-related antigens, including HBsAg (Thi et al., 2024). The Part 3 of the AB-729-001 trial delved into the therapeutic efficacy of imdusiran (Yuen et al., 2022a). Non-cirrhotic CHB patients were recruited for this study. They were administered AB-729 at doses of either 60 mg or 90 mg, with dosing intervals set as Q4W, every 8 weeks (Q8W), or every 12 weeks (Q12W). Among these groups, except for the group of HBV DNA-positive patients who were given tenofovir disoproxil fumarate on the first day, the other patients had achieved virological suppression after undergoing stable NA therapy. The trial results demonstrated that repeated dosing of imdusiran was generally well tolerated and safe. Across different dosing regimens, there were observable reductions in HBsAg levels. Notably, the extent of HBsAg reduction seemed to be independent of the dosage, the baseline HBeAg status, or the presence of baseline HBV DNA. At the Global Hepatitis Summit 2023 (GHS2023), Yuen et al. presented data on 9 CHB patients who had completed 48 weeks of imdusiran treatment (Yuen et al., 2023b). After 24 weeks of treatment, these patients met the protocol criteria for discontinuing NA therapy, which included having alanine aminotransferase (ALT) levels less than twice the upper limit of normal (ULN), undetectable HBV DNA, negative HBeAg status, and HBsAg levels less than 100 IU/mL in two consecutive visits. After discontinuing NA therapy, 78% (7 out of 9) of these patients maintained low HBV DNA levels, and their HBsAg levels remained below the baseline values (ranging from −0.8 to −1.6 log10 IU/mL). During the follow-up period, no adverse reactions were reported, and there were no occurrences of ALT flares. These findings imply that imdusiran holds promise in potentially achieving a functional cure for CHB.

RBD-1016 is a GalNAc-conjugated siRNA drug that targets the HBV X ORF (Li Q. et al., 2024). This drug is developed based on RNAi technology, which exerts inhibitory effects on the four transcripts of HBV. Moreover, it can effectively reduce the levels of HBsAg originating from both cccDNA and integrated HBV DNA. In the Phase I clinical trial (NCT05017116), treatment-naive or previously treated CHB patients without liver fibrosis or cirrhosis were enrolled (Seto et al., 2023). The drug was administered in two different regimens: a single-dose (SD) regimen and a multiple-dose (MD) regimen (administered on Day 1 and Day 29). The dosage was gradually escalated from 0.3 to 3.0 mg/kg. Throughout the study period, RBD-1016 was used in combination with NAs. The final results indicated that participants who received 3 mg/kg of RBD-1016 under the SD and MD regimens experienced a maximum mean reduction in serum HBsAg of 0.97 log_10_ IU/mL (at week 16) and 1.34 log_10_ IU/mL (at week 16), respectively. This reduction in HBsAg levels was sustained until the end of the study at week 24. Currently, RBD-1016 is being evaluated in global multi-center Phase II clinical trials. Additionally, clinical studies for another indication, hepatitis D, are also in progress.

HRS-5635 is a triantennary (GalNAc)_3_-conjugated siRNA drug. It precisely targets the HBV X gene. By doing so, it effectively inhibits the expression of HBV-related proteins, thus exerting potent antiviral effects. In 2023, this drug received approval to proceed with human clinical trials. Subsequently, in the first half of 2024, it advanced to Phase II clinical trials (NCT06425341). Presently, the Phase II clinical trial is actively recruiting patients. The primary objective of this trial is to comprehensively assess the safety and efficacy of HRS-5635 when used as a monotherapy or in combination with other therapeutic agents for the treatment of CHB.

TQA3038, an RNAi therapeutic agent crafted by Chia Tai Tianqing Pharmaceutical Group Co., Ltd., is designed for the treatment of CHB. This GalNAc-conjugated siRNA drug can accumulate in the liver and effectively block the replication of HBV. In the Phase I clinical trials conducted on healthy volunteers (NCT06085053), TQA3038 demonstrated favorable safety profiles and was well-tolerated. Moreover, its pharmacokinetic characteristics met expectations (Liu et al., 2024). Presently, a Phase Ib/IIa clinical trial of TQA3038 injection is actively being carried out among patients suffering from CHB.

### 6.3 miRNA

Unlike ASOs and siRNAs, miRNA-based therapeutics have demonstrated promising results in preclinical studies, though their clinical application remains in its early stages (Luna et al., 2016; Seyhan, 2024). Current research on miRNAs for treating CHB is primarily confined to laboratory investigations. Several miRNAs—including miR-122 (Bandiera et al., 2015; Luna et al., 2016; Manea et al., 2023), miR-146 (Wang and Tian, 2017; Wang and Li, 2018), and miR-101 (Wang and Tian, 2017; Wakasugi et al., 2018)—have been proven to regulate HBV replication. Currently, there are two principal strategies that guide the design of miRNA drugs: miRNA mimics and AntimiRs (Rupaimoole and Slack, 2017). miRNA mimics, such as artificial microRNAs (amiRNAs) (Li and Rana, 2014; van Rooij and Kauppinen, 2014), function analogously to endogenous miRNAs by targeting mRNAs to induce degradation or translational repression, thereby suppressing specific gene expression. AntimiRs (Stenvang et al., 2012; Hogan et al., 2014), such as miRNA inhibitors, employ sequences complementary to miRNAs to block their activity, modulating downstream gene expression for therapeutic benefit (Tang et al., 2017; Cannataro and Cione, 2023).

AmiRNA is a synthetically designed antisense sequence that targets specific genes by leveraging the natural miRNA biogenesis pathway and mechanism of action. Due to its inherent biological compatibility, amiRNA exhibits superior safety compared to other small nucleic acid therapeutics. It is typically delivered via viral vectors and enables sustained gene silencing in target tissues (Kotowska-Zimmer et al., 2021). Mao et al. employed the Invitrogen online tool Block-iT RNAi Designer (http://rnaidesigner.invitrogen.com/rnaiexpress) to screen 17 amiRNAs targeting conserved regions of the HBV genome (Mao et al., 2022). They compared the effects of three different tandem amiRNAs on HBV replication. Among these, amiRNA135 demonstrated robust inhibition across multiple HBV genotypes (A, B, C, D) and mutant isolates (Bm and Cm) *in vitro*. Furthermore, they constructed adeno-associated virus 8 (AAV-8) vectors as delivery systems. The results showed that AAV8-amiRNA135 could induce long-term suppression of HBV replication *in vivo*, with HBsAg and HBeAg levels remaining significantly reduced for up to 15 months in high-dose groups. Similarly, Maepa et al. developed anti-HBV artificial mono- and trimeric primary microRNAs (pri-miRs) using human pri-miR-31 as a scaffold (Maepa et al., 2017). These were packaged into self-complementary AAV8 (scAAV8) vectors for liver-specific delivery. Systemic administration of scAAV8-pri-miRs in HBV transgenic mice led to durable suppression of viral replication, highlighting the therapeutic potential of this approach.

AntimiRs are single-stranded oligonucleotides structurally analogous to ASOs, also referred to as antagomiRs. These molecules incorporate complementary sequences to target miRNAs and exert a functional blockade by forming stable duplexes, thereby sequestering the miRNA and preventing its interaction with endogenous mRNA targets (Rupaimoole and Slack, 2017). To enhance stability, antimiRs are typically chemically modified—commonly through PS modification, 2′-MOE modification, or LNA modification—to improve cellular uptake and ribonuclease resistance (Cannataro and Cione, 2023). Among therapeutic targets, miR-122 has emerged as a promising candidate for viral hepatitis therapy. Intriguingly, miR-122 exhibits differential regulatory effects on hepatitis virus replication: it promotes HCV replication while suppressing HBV replication (Song et al., 2015). This dual functionality positions miR-122 as both a potential miRNA mimic (for HBV inhibition) and an antagomir (for HCV suppression) (Thakral and Ghoshal, 2015). Capitalizing on this mechanism, Santaris Pharma A/S developed Miravirsen, an LNA-modified 15-nucleotide antimiR-122 oligonucleotide. Miravirsen binds mature miR-122 with high affinity, forming a stable heteroduplex that disrupts miR-122-mediated viral propagation. Currently, this drug has advanced to Phase II clinical trials for HCV therapy (Janssen et al., 2013).

## 7 Combination therapy

The goal of small nucleic acid drugs is to improve functional cure rates; however, this remains unattainable with conventional monotherapy. Although these drugs can achieve significant reductions in viral antigens, sustained clearance is rarely achieved when used alone. Future therapeutic strategies will likely require combinations of agents targeting distinct mechanisms: (1) viral replication suppression (e.g., HBV entry inhibitors (Urban et al., 2014; Kang and Syed, 2020), NAs, capsid assembly modulators (Nijampatnam and Liotta, 2019; Yang et al., 2019; Kum et al., 2023)); (2) antigen burden reduction (e.g., siRNAs, ASOs); and (3) immune response modulation (e.g., IFN-α, TLR agonists (Kayesh et al., 2021; Roca Suarez et al., 2024), therapeutic vaccines (Lang-Meli et al., 2021), monoclonal antibodies (Beretta and Mouquet, 2022), or checkpoint inhibitors (Fisicaro et al., 2018)). Long-term viral replication inhibitors may diminish the cccDNA reservoir, thereby reducing antigen load and potentially reversing immune exhaustion (Cornberg et al., 2020). Concurrently, immune modulators could amplify cellular immunity and restore pathogen-specific responses. Such combinatorial approaches are anticipated to enable durable viral suppression (Chen et al., 2007; Loglio et al., 2021; Lim et al., 2023). Currently, multiple clinical trials are underway to evaluate small nucleic acid drugs in multidrug regimens (Table 2). According to the disclosed clinical trial data, monotherapy and combination therapy show different treatment effects (Table 3).

**TABLE 2:** Combination/sequential therapy with small nucleic acid drugs.

Type	Agent	Category	Stage of development	Clinical trial number
Dual therapy combinations	VIR-2218+PEG IFNα	siRNA+PEG IFNα	Phase II	NCT05970289
	AB-729+vebicovir	siRNA+CAM	Phase II	NCT04820686
	JNJ-3989+NA	siRNA+nucleoside analogue	Phase I/II	NCT03365947
	GSK3228836+JNJ-3989	ASO+siRNA	Phase II	NCT06537414
	AB-729+AB-101	SiRNA+PD1 inhibitor	Preclinica	—
Triple therapy combinations	VIR-2218+VBI-2601+PEG IFNα	siRNA+therapeutic vaccine+PEG IFNα	Phase II	NCT06491563
	AB-729+PEG IFNα+NA	siRNA+PEG IFNα+nucleoside analogue	Phase IIa	NCT04980482
	JNJ-3989+PD1 inhibitor+NA	SiRNA+PD1 inhibitor+nucleoside analogue	Phase II	NCT05275023
	JNJ-3989+NA±JNJ-6379 (bersacapavir)	siRNA+nucleoside analogue±CAM	Phase II	NCT03982186
	GSK3228836+PEG IFNα+NA	ASO+PEG IFNα+nucleoside analogue	Phase IIb	NCT04676724
	VIR-2218+VIR-3434±PEG IFNα	siRNA+Monoclonal antibodies±PEG IFNα	Phase II	NCT04856085
	GS9688+nivolumab±VIR-2218	TLR-8 agonist+PD1 inhibitor±siRNA	Phase II	NCT04891770
	JNJ-3989+JNJ-64300535+NA	siRNA+therapeutic vaccine+nucleoside analogue	Phase I	NCT05123599
	GSK3228836+ETV±VE03702	ASO+nucleoside analogue±TLR-7/8 agonist	Preclinica	—

**TABLE 3:** Clinical trials of monotherapy and combination therapy.

Type	Agent	Trial phase &clinical trial number	Key focus & results
Monotherapy agents	GSK-3228836	Phase IIb (NCT04449029)	Evaluated efficacy/safety Results: 9%–10% of patients achieved undetectable HBsAg at 24 weeks. Better outcomes in patients with baseline HBsAg <3,000 IU/mL
	AHB-137	Phase I/IIa (NCT05717686, NCT06115993)	Focused on HBsAg reduction Results: 62% in the 300 mg group achieved HBsAg seroclearance by week 12, with some seroconversion
	VIR-2218	Phase I/II (NCT03672188)	Evaluated dose-dependent HBsAg reduction Results: 50% of patients had HBsAg <100 IU/mL; max reduction of 1.65 log IU/mL (200 mg group)
	JNJ-3989	Phase IIa (NCT03365947)	Investigated HBsAg reduction in CHB patients receiving NAs Results: 97.5% (≥100 mg) had ≥1 log10 reduction; 75% achieved HBsAg <100 IU/mL
		REEF-2 (NCT04129554)	Combined with NAs and CAMs Results: 71.1% achieved HBsAg <100 IU/mL at 48 weeks
	AB-729	AB-729-001 Trial	HBsAg reduction in patients on stable NA therapy Results: Sustained HBsAg declines; 78% maintained low HBV DNA after NA discontinuation
	RBD-1016	Phase I trial (NCT05017116)	Focused on HBsAg reduction Results: Maximum mean reduction of 1.34 log10 IU/mL in multiple-dose regimen
	HRS-5635	Phase II trial (NCT06425341)	Currently recruiting patients to assess HBsAg reduction as a primary endpoint
	TQA3038	Phase I trial (NCT06085053)	Evaluated safety
Combination therapy	VIR-2218 + PEG IFN-α	Phase II Trial(NCT03672188)	Combination therapy showed higher rates of HBsAg seroclearance compared to monotherapy
	JNJ-3989 + NAs	REEF-1, NCT03982186	Significant HBsAg reductions observed, though functional cure was not achieved

A phase 2 clinical trial (NCT03672188) has reported on the safety and antiviral activity of VIR-2218, either used alone or in combination with pegylated (PEG) IFN-α-2a, in patients suffering from CHB (Yuen et al., 2024). The participants in this trial were allocated into six groups. They received 200 mg of subcutaneous VIR-2218 Q4W. Some of these groups also received 180 μg of subcutaneous PEG IFN-α-2a on a weekly basis, and the treatment duration extended up to 48 weeks. Throughout the entire treatment period, all participants continued to undergo nucleoside or nucleotide reverse transcriptase inhibitor (NRTI) therapy. The trial results suggest that VIR-2218, whether administered as a monotherapy or in combination with PEG IFN-α-2a, is generally well-tolerated by the patients. When comparing the use of VIR-2218 or PEG IFN-α-2a alone with their combination, it was found that the combination therapy demonstrated higher rates of HBsAg seroclearance. Moreover, with a longer treatment duration, there was a more significant mean maximum reduction in HBsAg levels from the baseline. Specifically, among the participants who received the combination treatment of VIR-2218 and PEG IFN-α-2a, 11 achieved HBsAg seroclearance at some point during the trial. Of these 11 participants, 10 showed anti-HBsAg seropositivity. Additionally, 6 participants were able to maintain HBsAg seroclearance for up to 24 weeks after the completion of the treatment. In contrast, none of the participants treated with VIR-2218 monotherapy or those on the 12-week regimen of VIR-2218 plus PEG IFN-α-2a achieved HBsAg seroclearance. Overall, the findings of this phase 2 trial lend support to the continued development of VIR-2218 as a promising therapeutic option for patients with chronic HBV infection.

A Phase IIb clinical trial (NCT03982186) was carried out to assess the efficacy and safety of the siRNA JNJ-3989 and the capsid assembly modulator JNJ-6379 (bersacapavir) in combination with NAs. The REEF-1 trial recruited CHB patients aged between 18 and 65 years who were under continuous NA treatment throughout the treatment period. These patients were then subjected to different regimens combining JNJ-3989 and JNJ-6379 (Yuen et al., 2023a). Patients were randomly allocated to one of three groups: the JNJ-6379 (250 mg) dual-therapy group, the JNJ-3989 (40/100/200 mg) dual-therapy group, and the triple-therapy group (JNJ-6379 250 mg plus JNJ-3989 100 mg). By week 48, 47 patients fulfilled the criteria for discontinuing NA therapy. The highest proportion of patients meeting these criteria was observed in the JNJ-3989 (200mg) dual-therapy group, reaching 19%. The decline in HBsAg levels was dose-dependent on JNJ-3989. In the JNJ-3989 (200mg) dual-therapy group, as many as 75% of patients achieved HBsAg levels below 100 IU/mL. Although the mean HBsAg concentrations in all groups containing JNJ-3989 gradually rose during the follow-up period, their mean reductions from the baseline values at week 24 still exceeded 1 log_10_ IU/mL. Ultimately, the researchers inferred that the antiviral treatment combinations employed were inadequate to attain a functional cure for CHB. Nevertheless, a significant decrease in HBsAg levels was witnessed in most patients treated with JNJ-3989, which might offer a solid basis for immune modulation. The REEF-2 trial is a Phase IIb, double-blind, placebo-controlled, randomized study (NCT04129554) that involved 130 HBeAg-negative CHB patients on NA therapy (Agarwal et al., 2024). The patients were randomly assigned to either the treatment group (receiving subcutaneous JNJ-3989 at 200 mg every 4 weeks, oral JNJ-6379 at 250 mg once daily, and oral NA once daily) or the control group (receiving oral NA once daily). The treatment lasted for 48 weeks, followed by a 48-week follow-up period. After 48 weeks of treatment, 71.1% of patients in the treatment group achieved an HBsAg level of less than 100 IU/mL, with a group mean HBsAg reduction of 1.89 log_10_ IU/mL. At week 48 of the follow-up, 46.9% of patients in the treatment group still maintained HBsAg levels below 100 IU/mL, with a group mean HBsAg reduction of 1.46 log_10_ IU/mL. Although the combined treatment regimen failed to achieve a functional cure ultimately, it significantly lowered HBsAg levels, increased the proportion of patients with suppressed HBV DNA, and led to fewer and less severe post-treatment HBV DNA rebounds and ALT flares. These outcomes are crucial steps towards achieving a functional cure for CHB.

## 8 Conclusion

As our understanding of the HBV replication process and the host’s immune response deepens, remarkable advancements have been made in the treatment of CHB, uncovering a plethora of potential therapeutic targets. Nevertheless, currently, only specific patient cohorts can attain high functional cure rates through NAs and PEG IFN-α therapy. For patients with high baseline HBsAg levels, monotherapy with PEG IFN-α or its combination with NAs still faces considerable hurdles in achieving a cure. The intricate HBV replication cycle, the persistent presence of cccDNA within the host cell nucleus, and the continuous synthesis of HBsAg from the integrated HBV DNA in the host genome present formidable challenges to attaining a functional cure for CHB. Moreover, the host’s immune response exerts a profound influence on treatment outcomes. With the rapid development of chemical modification and liver-targeted delivery systems, small nucleic acid drugs are now regarded as the most promising candidates for achieving a functional cure. Owing to their distinctive mechanism of action, these drugs can interfere with the transcription process of HBV cccDNA, rendering the cccDNA in a “silent” state, and thus potentially leading to a functional cure for hepatitis B. They can also precisely target HBV RNA, effectively suppress viral replication, and lower viral antigen levels, thereby breaking immune tolerance. Additionally, small nucleic acid drugs possess high target specificity. They can be rationally designed according to the target gene sequences, and multi-target siRNA cocktail therapy can significantly mitigate the risk of drug resistance. Nonetheless, several issues remain to be resolved in the future, such as how to maintain the *in vivo* stability of small nucleic acid drugs, avoid immune reactions, and minimize off-target effects. Small nucleic acid drugs are anticipated to play a key role in the next era of CHB treatment. Given the difficulty of achieving a functional cure through monotherapy, future treatment strategies will likely involve combination therapies. The ability of small nucleic acid drugs to efficiently inhibit viral replication and reduce antigen levels makes them the foundation of combination therapy. They can be used in combination with other antiviral drugs and immunomodulators to reach the ultimate goal. However, further research is required to determine the optimal timing of combination therapy, the sequence of different drugs, the duration of treatment, and personalized treatment approaches. Based on the current clinical and pre-clinical data for small nucleic acid drugs, as well as the emergence of innovative drugs and combination therapies, it is believed that this emerging therapeutic strategy will further boost the cure rate of CHB, decrease the risk of cirrhosis and hepatocellular carcinoma, and bring the dawn of a functional cure for CHB patients worldwide.
